# How do people with rheumatoid arthritis experience participation in a smoking cessation trial: a qualitative study

**DOI:** 10.1080/17482631.2020.1725997

**Published:** 2020-02-11

**Authors:** Ida Kristiane Roelsgaard, Thordis Thomsen, Mikkel Østergaard, Anne Grete Semb, Lena Andersen, Bente Appel Esbensen

**Affiliations:** a Copenhagen Center for Arthritis Research (COPECARE), Center for Rheumatology and Spine Diseases, Centre for Head and Orthopaedics, Rigshospitalet, Glostrup, Denmark; b Herlev Anaesthesia Critical and Emergency Care Science Unit, ACES, Department of Anesthesiology, Copenhagen University Hospital, Herlev-Gentofte, Copenhagen, Denmark; c Department of Clinical Medicine, Faculty of Health and Medical Sciences, University of Copenhagen, Copenhagen, Denmark; d Preventive Cardio-Rheuma Clinic, Department of Rheumatology, Diakonhjemmet Hospital, Oslo, Norway; e The Danish Rheumatism Association, Gentofte, Denmark

**Keywords:** Smoking cessation, smoking cessation intervention, nicotine replacement therapy, smoking, rheumatoid arthritis, qualitative research, behaviour change

## Abstract

**Purpose**: The aim of this study was to gain more knowledge on how people with rheumatoid arthritis (RA) experienced participation in a randomized controlled trial (RCT) testing the effect of a smoking cessation intervention since this intervention have not been tested on an RA population before

**Methods**: We conducted a qualitative study with semi-structured individual interviews with 12 participants from the intervention group in the RCT.

**Results**: Through thematic analysis we identified four themes: ***Instilling hope for smoking cessation***, referring to the initial invitation to participate in the RCT; ***Various components of importance in the intervention***, referring to cooperation with the smoking cessation counsellor, improved carbon monoxide levels, fear of becoming addicted to nicotine replacement therapy, and suggestions for additional components in the intervention which could promote motivation; ***Breaking habits***, referring to ongoing reflection on quitting smoking; and ***Increased awareness of health, arthritis and smoking***, referring to the lack of information on smoking and RA from health professionals, and the impact of smoking on RA symptoms and overall health.

**Conclusion**: The results reflect the participants’ perspective on what is meaningful to them when trying to quit smoking and adds important knowledge to future smoking cessation studies in this patient group.

## Introduction

Smoking is one of the most significant modifiable environmental risk factors for rheumatoid arthritis (RA) (Manfredsdottir et al., 2006; Scott, Wolfe, & Huizinga, 2010). Studies suggest that 25–30% of people with RA in Denmark smoke (Loppenthin et al., 2015; Primdahl, Clausen, & Horslev-Petersen, 2013). This is almost twice as many as in the background population in Denmark (Sundhedsstyrelsen, 2018). Smoking may exacerbate the progression of RA and reduce the effect of antirheumatic treatment (Abhishek, Butt, Gadsby, Zhang, & Deighton, 2010; Chang et al., 2014; Soderlin, Petersson, & Geborek, 2012; Vittecoq, Richard, Banse, & Lequerre, 2018). Furthermore, smokers with RA experience higher disease activity compared to non-smokers with RA (Nyhall-Wahlin, Petersson, Nilsson, Jacobsson, & Turesson, 2009). This indicates that smoking cessation could be crucial for the treatment effect and disease outcomes of RA.

Successful long-term smoking cessation is usually achieved by several attempts of quitting before success (Chaiton et al., 2016; Tobacco Use and Dependence Clinical Practice Guideline Panel, 2000). Smoking cessation interventions have been demonstrated to be effective in healthy individuals, people who are hospitalized, and people with chronic obstructive pulmonary disease (COPD) and cardiovascular diseases (CVD) (Barth, Jacob, Daha, & Critchley, 2015; Rigotti, Clair, Munafo, & Stead, 2012; Stead et al., 2013; Stead & Lancaster, 2012; van Eerd, van der Meer, van Schayck, & Kotz, 2016). The interventions include behavioural support, nicotine replacement therapy (NRT), a combination of behavioural support and NRT, and prescription medication. Several Cochrane reviews conclude that the most effective smoking cessation intervention for both healthy individuals and people with COPD and CVD is behavioural support and NRT (Barth et al., 2015; Stead & Lancaster, 2012; van Eerd et al., 2016). We lack evidence regarding the optimal interventionintensity andduration required for smoking cessation(Stead & Lancaster, 2012). A recent review found that increased behavioural support increase the chance of successful smoking cessation by up to 20% (Stead, Koilpillai, & Lancaster, 2015). However, another study showed that, although brief advice for people with RA increased their awareness of the link between smoking and RA, it did not increase smoking cessation rates (Harris, Tweedie, White, & Samson, 2016). Studies of smoking cessation interventions for people with RA are sparse.

Currently we are carring out a randomized controlled trial (RCT) testing the effect of an intensive smoking cessation intervention on smoking cessation and disease activity in people with RA (Roelsgaard et al., 2017). The intervention consists of behavioural support and NRT to current smokers with RA (Roelsgaard et al., 2017). We wished to evaluate how participants experienced the intervention provided in the RCT and the experiences of smoking cessation. We wished to explore the patients’ experience of the intervention regardless of whether they stopped smoking or not. We were therefore eager to gain insight into the perspectives of both those who stopped smoking and those who did not stop smoking. Furthermore, we also wished to explore the patients’ knowledge of the detrimental effects of smoking on RA and its treatment. This knowledge can guide further development of smoking cessation interventions for these patients.

Therefore, the aim of this study was to gain more knowledge on how people with RA experienced participation in an RCT testing the effect of a smoking cessation intervention since this intervention have not been tested on an RA population before.

## Materials and methods

### Design and study setting

This study employed a descriptive qualitative design involving people with RA who had participated in an international RCT, that tested the effect of an intensive smoking cessation intervention on smoking cessation and disease activity in smokers with RA (NCT02901886) (Roelsgaard et al., 2017). The intervention arm consisted of a smoking cessation intervention with five smoking cessation counselling sessions combined with free NRT for six weeks. Furthermore, we measured carbon monoxide levels in exhaled breath at least one time during the intervention. The content of the counselling sessions is described in detail in Table I. The people in the control arm received standard care i.e., no smoking cessation counselling or NRT. All participants from the RCT had four follow-up visits within 58 weeks, with various measurements e.g., RA-specific outcomes, cardiovascular outcomes and patient-reported outcomes (e.g., pain, health-related quality of life, flare and disease activity).

**Table I.** The intervention in the RCT—consisting of five sessions with a smoking cessation counsellor in combination with nicotine replacement therapy

Meeting	Themes
1.	An introduction to the counselling course and preparation for smoking cessation, including the participant’s smoking status and their motivation for cessation. A diary is handed out to help the participant keep track of smoking pattern.
2.	Aims to prepare the participant for the three first days without smoking. A plan for nicotine replacement therapy (NRT) is made and the participant is given the agreed form of NRT. A diary is handed out to help the participant keep track of smoking pattern and NRT use.
3.	Aims to help the participant with issues concerning quitting smoking, including risk situations, relapse, rewards, social network and smoking stop. A diary is handed out to help the participant keep track of smoking pattern and NRT use.
4.	Includes maintaining motivation, physical activity, handling of stress and mood swings. A diary is handed out to help the participant keep track of smoking pattern and NRT use.
5. (final)	Includes continuing help with smoking cessation and preparation for the time after the intervention, phasing out NRT, and measurement of carbon monoxide in exhaled breath. A diary is handed out to help the participant keep track of smoking pattern and NRT use.

### Participants

Participants were recruited consecutively from the intervention group in the RCT after having completed both the intervention and the three months follow-up visit. Successful smoking cessation was not an inclusion criterion, hence both quitters and non-quitters were asked to participate. Eligible patients were informed about the project both orally and in writing, and invited to participate either by telephone or face-to-face at the Centre for Rheumatology and Spine Diseases outpatient clinic, Rigshospitalet. The median age of the participants was 62 years, 6 females and 6 males participated, and the median years of smoking was 46.5 years. Additional participant characteristics are described in Table II.

**Table II.** Participant characteristics

Characteristics	N = 12
Age, median (range)	62 (33–71)
Female, n (%)	6 (50)
Married/cohabiting, n (%)	10 (83.3)
Years of school attendance, median (range)	1 (0–5)
Current connection to labour market, n (%)	8 (66.7)
Duration of RA, median (range)	12 (2–28)
Fagerstöms Nicotine Dependence, median (range)	4.5 (2–8)
Current smoker, n (%)	10 (83.3)
Non-smokers, n (%)	2 (16.6)
Age at smoking debut, median (range)	14.5 (12–18)
Years of smoking, median (range)	46.5 (17–57)
Number of cigarettes per day, median (range)	17.5 (15–20)
Pack years, median (range)	39.5 (12.75–57)
Number of previous quitting attempts, median (range)	2.5 (0–8)

### Data collection

In total, 12 patients accepted the invitation to participate in the study, and agreed to participate. Individual, semi-structured interviews were performed at the Centre for Rheumatology and Spine Diseases, Rigshospitalet, Glostrup between February and July 2018 (lasting between 45 and 75 minutes, mean 60 minutes) by BAE, who had no prior involvement with or knowledge of the participants.

Semi-structured individual interviews were conducted using an interview guide that was developed to elicit the participants’ experiences of participating in the RCT and the smoking cessation intervention (Table III).

**Table III.** The interview guide

Opening question
*What do you know about smoking and rheumatoid arthritis?*
**1: Smoking history**
*Please, tell me about your experiences of smoking?*
**2: Smoking and arthritis**
*Which considerations do you have about smoking and your health now and in the future?*
**3: Participation in the randomized controlled trial**
*What were your reasons for participating in the study?*
**4: Content in the intervention**
*How did you experience the smoking cessation sessions?*
**5: Motivation and barriers**
*What motivation and barriers have you experienced in the process?*
**Closure**
*If a further study were to be undertaken, what ddo you think it should include?*

The interview guide was based on the elements of the intervention in the RCT and previous studies of smoking cessation interventions, and motivation and barriers to smoking cessation (Aimer et al., 2018, 2015). The interview guide helped the interviewer (BAE) to ensure that coverage of all desired areas related to the aim of the study.

### Patient research partner

A PRP with RA, who was a former smoker, was actively involved throughout the conduct of the study according to the principles of respect and equality between PRP and researchers (de Wit et al., 2011; Hewlett et al., 2006; Trivedi & Wykes, 2002). The PRP participated in developing the interview guide, interpretation of the analysis and results as well as commented and approved the final version of the manuscript.

### Analysis

The interviews were recorded digitally and transcribed verbatim. All transcripts were uploaded to NVivo (version 11, QSR International) for a structured analysis. Latent thematic analysis was applied, as described by Braun and Clarke (Braun & Clarke, 2006), by continuously asking: “In what way did the participants talk about their experience of the randomized controlled trial and intensive smoking cessation intervention?” The approach was inductive, which entailed that the themes were strongly connected to the raw data. The analysis was a six-phase process (Braun & Clarke, 2006).

Initially [1], we became familiarized with data by listening to the interviews and comparing them to the transcribed interviews.Then [2], we generated initial codes based on the interview guide, and [3] organized the codes and searched for initial themes. Thereafter [4], we reviewed the themes by comparing them with one another. Then [5], we defined, redefined and labelled the themes, and finally [6] we wrote up the results and included vivid examples from the data-extract (Braun & Clarke, 2006). The initial analysis was performed by the first author (IKR) in close collaboration with TT, BAE and a patient research partner (PRP). During the analysis, we continuously supplemented and contested each other’s statements to ensure the results were grounded in the participants’ experiences.

### Ethics

The study was approved by the Danish Data Protection Agency (RH-2017-274, I-suite 05814) and carried out according to the principles of the Helsinki Declaration. Participants provided signed informed consent prior to the interviews. All data were treated confidentially and stored according to current legislation.

## Results

From the interviews, we identified four themes and six sub-themes through thematic analysis (Table IV). The aim of this study was to gain knowledge on how the participants experienced participation in the RCT. From the analysis we learned that the patients wanted to stop smoking and the invitation to participate in the RCT induced hope for successful smoking cessation. They experienced the different components in the intervention to be important for smoking cessation, however, smoking cessation nevertheless proved difficult. The patients expressed that health professionals should inform patients about the detrimental effects of smoking on RA.

**Table IV.** Themes identified in the analysis

Theme	Subtheme
1. Instilling hope for smoking cessation	
2. Various components of importance in the intervention	*2a. Having a fellow traveller on the road to smoking cessation*
	*2b. Carbon monoxide levels in exhaled breath as a motivational factor*
	*2c. Apprehension—fear of a new dependence*
	*2d. Suggestions for additional elements in the intervention*
3. Breaking habits	
4. Increased awareness of health, arthritis and smoking	*4a. Why wasn’t I told?*
	*4b. The effect of smoking cessation on arthritis and general health*

### Theme 1: instilling hope for smoking cessation

The participants saw the project as an opportunity to quit smoking and the project instilled hope of achieving smoking cessation. The participants felt it was positive that health staff from the Department of Rheumatology were interested in helping them stop smoking.

There were participants who were happy to be part of the intervention group of the RCT, as it gave them extra motivation for smoking cessation.

*“YES, I thought, now I have all the opportunities, and now you really have to grab it, and just do it, and then we we’ll see how it goes … ”* Interview J.

One said that he would have preferred to be in the control group, so he could continue as usual and take “the easy way”. Some did not think they would be able to give up smoking if they were in the control group, because they would be on their own without support; they felt that, with less focus on their smoking consumption, their motivation would be challenged.

### Theme 2: various components of importance in the intervention

#### Sub-theme 2a: having a fellow traveller on the road to smoking cessation

The participants had an experience of being greeted positively and not being looked down upon during the intervention. This was highlighted as very important, as otherwise they would just have backed out of the project—as they expressed it. Not giving up on smoking during the trial was described it this:

*“Yes, I was embarrassed that I hadn’t quit, but I wasn’t … looked down upon for that reason. They were sweet and nice towards me and encouraged me, even though I had not stopped, which I think was nice.”* Interview A.

The positive attitude of the smoking cessation counsellor was emphasized and this made it easier to get back on track if they had a setback. The principles of the intervention were perceived as non-judgemental, which motivated participants to make an effort and attend the following counselling session. The length of the counselling sessions was considered adequate. The participants experienced that they were very engaged while the intervention was ongoing and that it was reassuring to know one had an appointment with the smoking cessation counsellor in the future if there had been set-backs in the smoking cessation process.

The diaries the participants were given to note their tobacco and NRT consumption, were considered helpful, both in the beginning and when the smoking cessation went well. However, the diaries became a burden when giving up smoking was not going well, because set-backs or increases in cigarette consumption were clearly documented.

*“Yes, yes, but there is also the thing, that it gets to you at home when you know that in a few weeks you will have to go there and be account for it again, and hand in your written notes of how many you have smoked … Yes, but I actually think it is a good thing that every evening when filling out the notes you are confronted with how it went, because otherwise you wouldn’t count … ”* Interview L.

#### Sub-theme 2b: carbon monoxide levels as a motivational factor

It was motivating to see carbon monoxide levels improving. The benefits of smoking cessation became tangible as the participants were not necessarily able to feel any effects of the smoking cessation or reduction of cigarette consumption on their health or RA. It was considered satisfying to follow the development themselves and the carbon monoxide results motivated their next attempt to give up smoking because they had seen how quickly the numbers improved.

*“Well, I was VERY impressed. Everything was improved. That made me really happy and I also thought: ‘This here can get you to continue’.”* Interview A

#### Sub-theme 2c: apprehension—fear of a new dependence

As part of the intervention, the participants were offered NRT. Some participants were afraid of becoming addicted to NRT and therefore were reluctant to use it.

*“ … And I think it is because the nicotine, I have not had the nicotine for almost four months now … And I think that was what I was most afraid of, which I have convinced myself to believe … That I was most afraid of the nicotine … I was afraid of the addiction.”* Interview J.

There were participants who tried different variants of NRT. Some described that they got unwell with the chewing gum and that the mouth spray tasted bad. Some of the participants did not experience any effects of the NRT. However, the participants ultimately settled on a variant of NRT that suited them.

#### Sub-theme 2d: suggestions for additional elements in the intervention

The participants provided ideas for further development of the intervention, for example, the inclusion of text messages and emails. Personalized messages could boost motivation because one would be reminded of the smoking cessation more frequently.

Participants varied as to whether they would prefer individual or group sessions. The advantage of group sessions could be inspiration and ideas from other group members, while individual sessions meant that you had to do the “project” all by yourself.

Opinions also varied regarding the potential benefit of having a partner participate in the intervention with you. On the positive side, this would allow participants to compete with their partner, which might increase motivation. Negatives could include the effect if the partner did not wholeheartedly go along with the intervention or interfered too much. Finally, some participants called for more counselling sessions in order to increase their success in quitting smoking.

*“I just think that there could have been a few more conversations … Yes, then I think that I could have quit completely … Had there been a few more conversations then I am pretty sure it would have motivated me to stop completely … .”* Interview C.

### Theme 3: breaking habits

Those who did not give up smoking described that their participation in the smoking cessation counselling sessions made them more aware of their smoking habits during and after the intervention.

*“Ohh, some more focus on that. And some more of, what is it called … That the cigarette you just smoked, you smoked automatically, now I’m more focused on not smoking on autopilot … So, now I’m more aware of the unnecessary cigarettes—if you can use a stupid term like that, so yes.”* Interview B

Participants slowly developed other habits, which they linked with the smoking cessation counselling. For example, if one could not see the cigarettes then one might forget them for a while. Some described that they had an improved insight into themselves in relation to risk situations and that this was helpful in the smoking cessation process.

*“Yes, but it is about getting to know myself in these situations and to get a handle on what exactly are the situations where I am emotionally pressured and remain focused on giving up smoking.”* Interview H.

### Theme 4: increased awareness of health, arthritis and smoking

#### Sub-theme 4a: why wasn’t I told?

Some of the participants had spoken to their rheumatologist about the negative side effects of smoking in relation to arthritis. None of the participants had spoken to a nurse about smoking and RA. The participants expressed that they needed more education on the link between smoking and RA and support for smoking cessation was brought up more regularly by both rheumatologists and nurses, specifically the effects smoking has on RA. Some were aware that smoking and RA were a negative combination, while others did not link RA and smoking. They wanted more knowledge about when they would be able to see improvement in their RA symptoms after smoking cessation.

*“The only thing I’ve never really had any information about is what smoking does to rheumatoid arthritis. I’ve never really got to know about that”*. Interview A

#### Sub-theme 4b: the effect of smoking cessation on arthritis and general health

The participants did not experienced improvement in their RA disease activity subsequent to smoking cessation or reduced cigarette consumption.

*“It is really hard to measure. That is, when you are already sick. Is it the disease or is it the smoking or what is it that makes you feel worse or better? … Because the arthritis goes up and down like crazy … Yes. It is strange. In the evening or in the morning I can feel really good. And then in the afternoon I can’t walk … Oh, yes, the ever-changing disease. It’s up and down, you know”* Interview D.

There were participants who felt a exacerbation in the RA after quitting andcould not help connecting the smoking cessation with the worsening of RA, but nevertheless knew that it was unlikely that the two things were related. It was disappointing as smoking had always been portrayed as negative by the rheumatologist, resulting in the feeling of solely responsibility for the aggressive disease activity. It was described that the rheumatologist had promised that the RA would improve if one stopped smoking.

*“Yes, in some ways it is nice, but on the other hand I usually like to say that none of my doctors have experienced having rheumatoid arthritis … but he can still not predict when I will get better … He promised me that if I gave up smoking then my arthritis would get better. ”* Interview J.

Some experienced improved health after giving up smoking or cutting back on cigarettes and described that they felt healthier. This feeling was mainly due to being able to breathe more easily and that was the most motivating part of the smoking cessation process.

*“I mean, yes that was nice. I mean it was lovely, you know. I could feel, apart from my rheumatoid arthritis that I felt an improvement in my health, you know. Like better breathing and a feeling of being a bit healthier. ”* Interview E.

## Discussion

The results of this study highlight that the offer of an intensive smoking cessation intervention was meaningful to people with RA. All the elements of the intervention—from the initial information about the study, the randomization, the different elements of the intervention, including motivational interviewing, diaries, NRT, and carbon monoxide measurements—were experienced as relevant by the patients. Overall, the participants expressed a positive attitude to the trial and the intervention because it instilled a hope for possible smoking cessation.

Participating in the intervention was considered to be a positive and motivational experience, because of the motivation generated with the smoking cessation counsellor and because carbon monoxide levels were monitored. The intervention in the RCT study (Roelsgaard et al., 2017) aimed to help people identify possible barriers to smoking cessation, and explore and overcome these barriers by focusing on something positive through motivational interviewing (MI) (Miller, 2002). *Motivational* is the keyword in MI, the concept on which the counselling sessions were based. This ensured that a positive approach was taken to the patients. The motivational factors could be both external and internal. External motivators included the interaction with the smoking cessation counsellor, monitoring of carbon monoxide levels, accountability through the diaries and NRT (which, for some, was considered to be a barrier). Internal motivators were the exploration of their ambivalence towards smoking cessation and the conscious decision to stop smoking (Miller, 2002). This is supported by a Cochrane review, demonstrating that motivational interviewing provided by general practitioners or by trained counsellors is more effective in helping smokers to quit smoking than is brief advice (Lindson-Hawley, Thompson, & Begh, 2015). Even if most of the participants in the current study did not succeed in quitting, they described the counselling sessions as motivating both during the intervention and for future quit attempts. This suggested that that the intervention positively influenced the participants’ awareness of their smoking habits even though they did not stop smoking. This could potentially improve their chances of a successful smoking cessation in the future, due to an increased awareness of the process, including barriers and risk situations, related to smoking cessation.

The participants expressed a need for more intensive smoking cessation support, for example more counselling sessions, emails and or SMS support. Some participants specifically expressed a need for an increased number of counselling sessions to quit smoking. This could be explained by a wish for more responsibility from the smoking cessation counsellor, as some expressed that they felt highly committed to the counsellor. However, the wish for more sessions is more likely to be an expression of not wanting to be “left alone” on the journey to smoking cessation—as also expressed through participants’ appreciation of having a fellow traveller on the journey to cessation. A higher amount of support was investigated in a pilot RCT by Aimer et al., who examined a tailored 3-month smoking cessation intervention consisting of behavioural support, email consultations and NRT (Aimer et al., 2017). The quit rates at 6-months follow-up were relatively high (overall 24%) (Aimer et al., 2017). NRT and brief advice were also provided to people in the control group, possibly explaining the high quit rates in the control group. The different wishes and needs among the participants in our current study and Aimer’s study could be helpful in designing future smoking cessation interventions for people with RA. These studies indicate that the interventions lasting longer than the intervention offered to participants in the current study should be tested.

All participants, in the intervention and control groups, had their carbon monoxide level measured at baseline, throughout the intervention and at follow-up. It was a general perception among the participants that they were motivated by the ongoing monitoring of carbon monoxide levels. The participants were enthralled by the immediate improvement in carbon monoxide levels, even if they could not feel the effect in their bodies. Monitoring of carbon monoxide levels in smoking cessation studies has previously been demonstrated to be motivational and has been reported in RCTs that tested carbon monoxide levels as a support and motivation for smoking cessation (Sejourne et al., 2010; Shahab, West, & McNeill, 2011). Carbon monoxide measurements therefore appear to be an important tool for sustaining motivation.

Some participants were fearful of becoming addicted to NRT. They feared that their smoking addiction would be replaced by an addiction to NRT. Some also had not experienced any effect of the NRT during previous quit attempts and therefore did not wish to try it again. It appeared that some participants wished to go “cold turkey” to avoid a new addiction to NRT, as also described in a qualitative study based on an internet forum for smoking cessation (Kurko, Linden, Kolstela, Pietila, & Airaksinen, 2015). In that study, participants expressed a negative attitude towards NRT, as they believed its use maintained tobacco dependence (Kurko et al., 2015). Apprehension towards the use of NRT may be a substantial barrier to successful smoking cessation. However, there is compelling evidence that NRT increases the chances of long-term smoking cessation by 50–70% (Silla, Beard, & Shahab, 2014; Stead et al., 2012). We therefore consider it crucial that smoking cessation counsellors educate smokers about NRT and encourage its use. In future studies, the effect of other types of pharmacotherapy, such as bupropion, varenicline and cytisine should therefore be examined in people with RA who smokes (Cahill, Stevens, Perera, & Lancaster, 2013).

Reflections on the association between smoking and RA varied among our participants. Many did not believe there was any connection between the two and felt no improvement in their RA after having cut down or completely quitting smoking. We do not know the time period from smoking cessation to a possible improvement of RA symptoms. Therefore, we could not inform patients of what to expect. To our surprise, the participants did not mention their RA disease much in the interviews which could be explained by a possible lack of knowledge about the link between smoking and RA. Qualitative studies have previously identified that one of the main barriers to smoking cessation in patients with RA and systemic lupus erythematosus was lack of knowledge of the links between smoking and RA (Aimer et al., 2015; Gath, Stamp, Aimer, Stebbings, & Treharne, 2018; Wattiaux et al., 2019). In those studies, patients expressed that they had not received information from their rheumatologist about the association between smoking and RA (Aimer et al., 2015; Wattiaux et al., 2019). However, awareness of the effects of smoking on RA could be motivate smoking cessation (Gath et al., 2018). The participants in our study asked for more information from health professionals about smoking, smoking cessation and the connection to RA. We do not know from our study to what degree participants were informed about the relation between RA and smoking. In Denmark, health professionals are obliged to talk to their patients about smoking and its negative impact. It remains unclear to what degree this is actually done. Nevertheless, the lack of awareness about the risks of smoking and RA is important knowledge, which can inform future planning.

There are several strengths of the current study. Firstly, the same person interviewed all the participants, ensuring homogeneity throughout the interviews. Furthermore, the interviewer was not involved in the intervention of the trial and had not previously been in contact with the participants, thereby reducing the risk of bias and ensuring the external validity of the study. Secondly, there were equal numbers of male and female participants, whereas most studies including people with RA show a higher proportion of women (as RA is more frequent in women). Finally, all the invited patients agreed to participate in this qualitative study. This is particularly interesting because some of those who accepted to participate did not quit smoking. Nevertheless, they were willing to share their experiences, which was of great importance,because they had perceived participation in the RCT to be a positive experience.

It could be argued that a limitation of this study was the inclusion of participants only from the intervention group (which was our aim). They were all positive about the project and felt that they had received help and support during their quit attempt. This might not have been the case had we included patients from the control group, however we wish to evaluate the intervention as well.

The results of the current study increase our insight into patients’ experiences of an intensive smoking cessation intervention. Patients were, overall, content with participating in the RCT and the intervention. The results suggest that gaining the patients’ trust is an important factor in their motivation to stop smoking. Even though several did not quit smoking, they portrayed the RCT and intervention as a positive experience that gave them tools for future smoking cessation attempts. The participants were motivated to stop smoking but most did not manage to quit. This could imply that motivation alone is not enough to succeed and that factors such as nicotine addiction and the fear of becoming addicted to NRT may be a strong barriers to smoking cessation.

In conclusion, the patients called for more focus by health professionals on smoking cessation. Smoking cessation counselling should be offered at the clinics where the patients are treated for RA. Smoking cessation is complex and the participants in our study encouraged health professionals to address and talk more about smoking and smoking cessation with patients. Future clinical studies that would test various types of smoking cessation interventions should include more counselling sessions and be of a longer duration, in order to improve the chances of quitting smoking among people with RA.

## Data Availability

The data that support the findings of this study are available upon reasonable request from the corresponding author, IKR. The data are not publicly available due to restrictions regarding information that could compromise the privacy of research participants. Data is available until 31/05/2024 where it should be destroyed according to the approval from the Danish Data Protection Agency (ID number 05814/RH-2017-274).
